# Osteosarcoma of the jaw – experience at the Medical University Vienna and comparative study with international tumor registries

**DOI:** 10.6061/clinics/2019/e701

**Published:** 2019-04-16

**Authors:** Christina Eder-Czembirek, Doris Moser, Simone Holawe, Thomas Brodowicz, Jutta Ries, Irene Sulzbacher, Edgar Selzer

**Affiliations:** I Department of Cranio, Maxillofacial and Oral Surgery, Medical University Vienna, Vienna, Austria; II Department of Internal Medicine I, Medical University of Vienna, Vienna, Austria; III Department of Oral and Maxillofacial Surgery, Erlangen University Hospital, Erlangen, Germany; IV Clinical Institute of Pathology, Medical University of Vienna, Vienna, Austria; V University Clinic of Radiotherapy, Medical University of Vienna, Vienna, Austria

**Keywords:** Osteosarcoma, Jaw, MAGE-A, Immunohistochemistry

## Abstract

**OBJECTIVES:**

Osteosarcoma of the jaw (OSAJ) is fundamentally different in clinical practice from its peripheral counterparts. Studies are difficult to conduct due to low incidence rates. The primary aim of this study was to provide for the first time a comprehensive retrospective analysis of the treatment concepts and outcome data of OSAJ patients treated at the University Hospital Vienna and to compare these with two recently published studies on OSAJ. The clinical study was accompanied by a biomarker study investigating the prognostic relevance of melanoma-associated antigen-A (MAGE-A) in OSAJ specimens.

**METHOD:**

Eighteen patients were included, and their outcomes were compared to published data. Immunohistochemistry was performed with mouse monoclonal antibodies against MAGE-A. Survival rates were estimated by the Kaplan-Meyer method. The log-rank test was used to analyze potential prognostic parameters. Fisher's exact test was performed to define the significant differences between the survival rates of the current study and the DOESAK registry.

**RESULTS:**

Disease-specific survival was 93.8% after five and 56.3% after ten years. The development of metastases (*p*=0.033) or relapse (*p*=0.037) was associated with worsened outcomes in our group as well as in the comparative group. Despite the different treatment concepts of the study groups, survival rates were comparable. MAGE-A failed to show prognostic relevance for OSAJ patients.

**CONCLUSIONS:**

Uncertainties about the optimal treatment strategies of OSAJ patients will currently remain. Thus, prospective studies of OSAJ are needed but are only feasible in a multicenter study setting, conducted over a prolonged time period.

## INTRODUCTION

Osteosarcoma (OSA) belongs to the most common primary malignant tumors of the bone, of which approximately 6-7% are primarily localized in the jaw bones 1-3. Approximately 5% of OSAs are thought to be secondary malignancies induced by exposure to ionizing irradiation 4-6. The reported incidence of radiation-induced OSAs is approximately 0.8% 5.

Unfortunately, comprehensive studies of osteosarcomas of the jaw (OSAJs) are difficult to conduct due to the low incidence (2 cases per 10 million). Thus, OSAJs are often subject to nonuniform treatment modalities. These strategies are often adapted from the treatment concepts of OSA of the long bones and craniofacial OSAs 3,7-10. No particular pathohistological, radiological or immunohistochemical differences between OSAJ and OSA of the peripheral bone (OSAP) have been described until now. However, OSAJ shows several clinical features distinct from those of OSAP. For example, patients with OSAJ are, on average, approximately two decades older, and they tend to have a lower risk for hematologic dissemination, which is associated with higher survival rates 3.

The primary aim of this study was to provide for the first time a comprehensive, retrospective analysis of the treatment concepts and outcome data of OSAJ patients treated at the Department of Cranio-, Maxillofacial and Oral Surgery at the University of Vienna from 1995 until 2015. Additionally, we sought to compare these results with data published by Baumhoer et al. 1 from the DOESAK (German-Swiss-Austrian Workgroup on Maxillofacial Tumors) tumor registry (https://doesak.med.uni-rostock.de/) and Lee et al. 3 who published data derived from the SEER (population-based US Surveillance, Epidemiology and End Results) cancer registry. As the Institute of Pathology at the Medical University of Vienna serves as a reference center for Austria, no specimens from our clinic have been deposited at the DOESAK registry.

Furthermore, we conducted an accompanying biomarker study investigating the expression of the melanoma-associated antigen-A (MAGE-A) family in OSAJ specimens. MAGEs, which are recognized by T-cells, are typically not expressed in normal tissues except for the testis, in which spermatogonia and spermatocytes are MAGE-A positive 11. The potential prognostic relevance of MAGE-A was demonstrated by several investigators 12-18. These publications showed a correlation of MAGE-A expression and the potential for the development of distant metastasis in various tumors. In particular, the expression of MAGE-A1, A3, A4, A6 and A12 isoforms are of interest because they were overexpressed in malignancies compared to levels in healthy tissues 11. Until now, little was known about the expression, function and potential therapeutic relevance of the MAGE-A family of proteins in OSA, although it has been suggested 19 that this antigen might serve as a potential target for adoptive immunotherapy 19-22. MAGE-A expression was therefore evaluated to assess a possible correlation with the malignant potential of OSAJs.

## MATERIALS AND METHODS

### Patients

Eligible participant for this retrospective study were patients who were treated for histopathologically proven OSAJ. We only included patients with complete documentation and follow-up. Patients with OSA of the cranial bones or other primary sites of the viscerocranial bones or OSA of the external skeleton were excluded. The observation period encompassed 20 years (1995-2015).

### Treatment

As OSAJ is rare and is clinically as well as therapeutically distinct from OSAP, no consensus about treatment recommendations exists 3,23. In most patients, primary surgery was performed with the intent to achieve clear resection margins if this procedure was deemed compatible with the preservation of essential organ function and/or quality of life. A wide resection was defined as no detectable tumor cells and indifferent tissue between tumor and resection margin regardless of distance, whereas (focal) marginal resection was characterized by the presence of reactively transformed tissue between the tumor and resection margin of any latitude. Neoadjuvant treatment consisted of chemotherapy regimens according to EURAMOS-1 24 or EURO-B.O.S.S. (EUROpean Bone Over 40 Sarcoma Study) protocols. Radiotherapy was indicated only in cases with primarily inoperable disease or local recurrences but not routinely in combination with surgery, in contrast to the procedure described in the study by Baumhoer et al. 1.

### Immunohistochemistry

**Figure 1:**
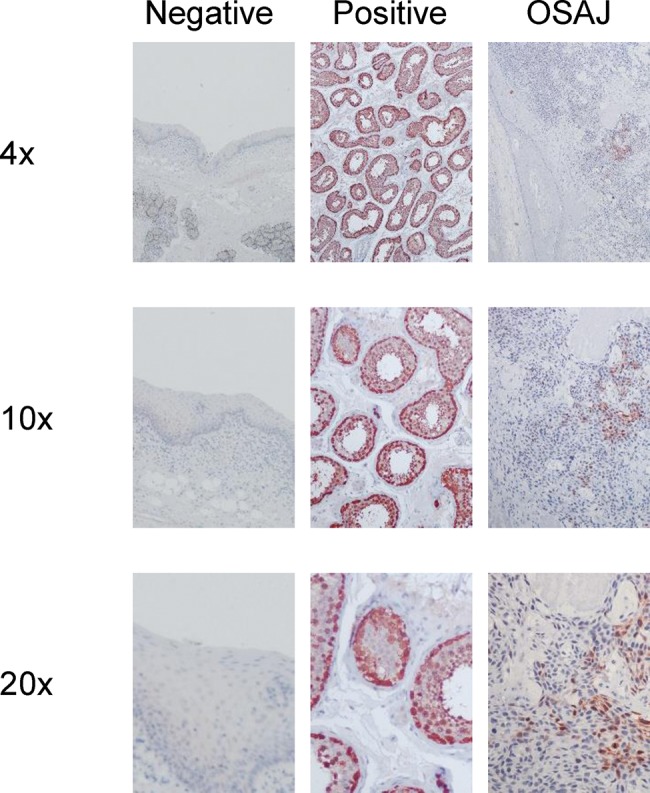
Immunohistochemical staining with MAGE-A Mab 6C1 (Dako). Left column: mucosal sections were used as negative controls; middle column: testis tissue sections were used as positive controls; right column: representative sections of OSAJ tissues; each at 4x, 10x and 20x magnification.

We performed immunohistochemistry (IHC) with mouse monoclonal antibodies against human MAGE-A (clone 6C1, anti-MAGE-A1, Santa Cruz Biotechnology, Inc., Dallas, Texas); Mab 57B anti-MAGE-A3 protein, which was kindly provided by C. Spagnoli, Basel, Switzerland). For representative images (Figure 1). In fact, most of the known MAGE-A family members are recognized by clone 6C1 25, while in tissue sections, Mab 57B primarily detects MAGE-A4 26. Sections (4 μm) of formalin-fixed and paraffin-embedded tissues were used for IHC analysis. Deparaffinization, antigen retrieval, and blocking of nonspecific binding were performed according to standard procedures described by the manufacturer. Tissue sections were incubated overnight at 4°C with the primary antibody (1:50 dilution of Mab 6C1 and 1:20 dilution of Mab 57B), followed by incubation with a polyclonal rabbit anti-mouse antibody (1:200, Dako Denmark A/S, Glostrup, Denmark) and subsequent immunohistochemical reaction with a streptavidin binding system (Dako Denmark A/S). The peroxidase reaction product was visualized by AEC+ high substrate chromogen stain (Dako Denmark A/S). After counterstaining with hematoxylin, slides were assessed by a pathologist. For each section, three regions of interest (∼450 to 550 cells) were defined, and the positively stained cells were counted under a microscope (magnification 20x). Healthy mucosal sections served as negative controls, and testis tissue served as positive controls for MAGE expression. Both nuclear and cytoplasmic staining were regarded as positive for MAGE-A expression. Staining patterns as well as the intensity of the staining were documented (0: no staining, 1: slight staining, 2: medium staining, 3: strong staining).

### Data Acquisition and Statistical Analysis

The authors reviewed the patient's individual institutional charts and directly contacted the Austrian National Register. Data obtained from medical records included patient age, gender, site, treatment modality, tumor grade and histological subtype. Demographic data are presented using the median, minimum and maximum values unless otherwise stated. Descriptive statistics were performed to define the relevant characteristics and their manifestation. A comparison of the proportions for the variables of interest of the study group *versus* published data was made using Fisher's exact test.

The following potential clinical and histopathological as well as immunohistochemical prognostic parameters were collected: presence or absence of metastatic disease at the time of diagnosis, occurrence of local or metastatic disease during follow-up, response to neoadjuvant chemotherapy, grading, resection margins and MAGE-A expression. MAGE-A expression was defined as either negative or positive without defining cut-off levels. Clinical, pathological parameters and MAGE-A expression were correlated with overall survival. Estimates of overall survival (OS) and disease-specific survival (DSS) and calculation of survival rates were derived by the Kaplan-Meier method. The relation between each variable and OSAJ was tested using the Cox proportional hazard model. The log-rank test (Mantel-Cox) was used for analysis of the significance of differences between the DSS rates of this study and of the DOESAK group (data shown in Table 2). *p-*values <0.05 were assumed to denote a statistically significant difference. Statistical analyses were performed with SPSS 24.0 (SPSS Inc., Chicago, IL, USA).

## RESULTS

We included 18 patients with OSAJs in this analysis and compared the results with the largest published datasets known. These were the results of the DOESAK registry group 1 and the results based on the SEER tumor database 3. Demographic data and a descriptive analysis are shown in Table 1. No significant differences between most of the characteristics compared were found except for type of the primary treatment modality and tumor grade. Baumhoer et al. 1 as well as Lee et al. 3 described a significantly higher percentage of exclusively surgical procedures for the treatment of OSAJ as well as a significantly higher incidence for low-grade OSAJ. The mortality in our study group was 22% (4/18 patients) compared to 28% in the DOESAK registry cohort.

The median follow-up in our collective was 26.0 (range 0.7-144.1) months. Four patients (22%) developed metastases, but only one patient had metastatic disease to the lung at the time of initial diagnosis. The median time for the development of metastasis was 14.8 months.

Wide resection margins were achieved in 62.5% of the operated cases. We did not attempt to further analyze the potential association of the width of resection margins to outcome due to the small number of patients in our study. Furthermore, the presence of close margins has not been found to be associated with local control in conventional OSA 27. Focal marginal resection was documented in our collective in 6 out of 16 (37.5%) operated patients. However, no attempts were made to further extend the surgery in any of these patients. Six patients (33%) developed a locoregional relapse, three of whom (12.5%) had wide resection. The median time to locoregional recurrent disease was 6.6 months.

Two of 18 (11.1%) patients who received chemotherapy without surgery did not achieve a complete response. Among those patients who received preoperative chemotherapy, only one of 5 patients showed a partial response towards chemotherapy.

**Figure 2:**
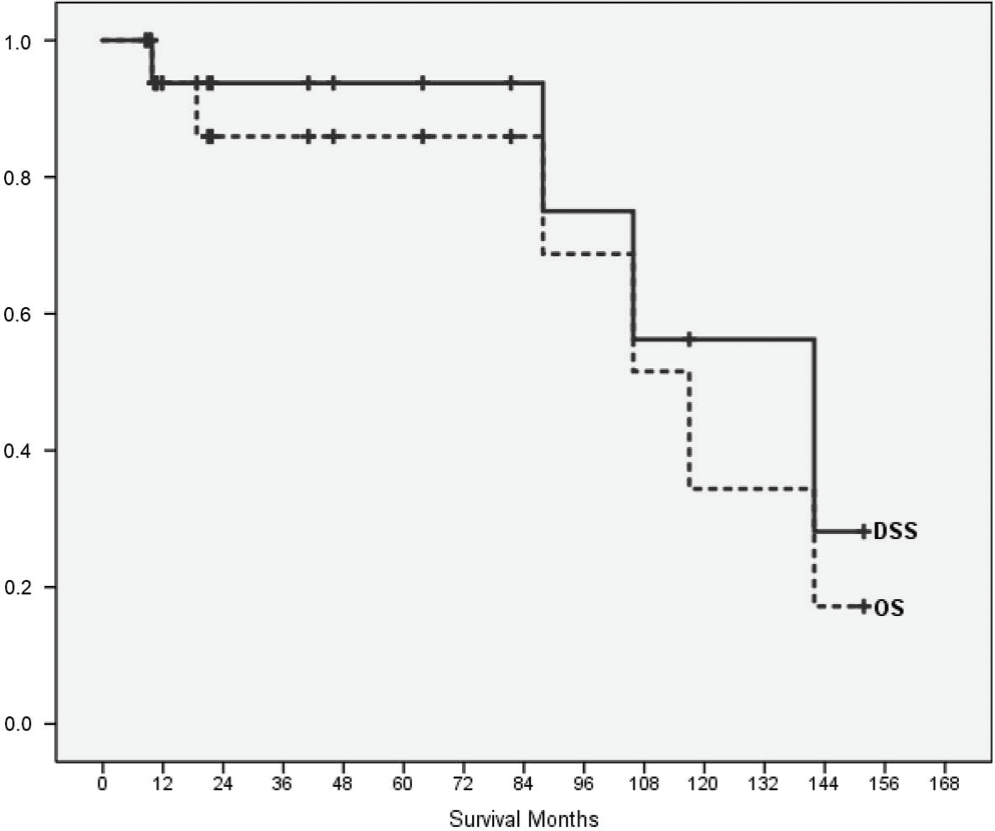
Kaplan-Meier curves for OSAJ OS and DSS rates of the study collective.

Kaplan-Meier estimates were calculated for OS and DSS (Figure 2). The median OS was 117.0 months (SE 16.3; CI 95% 85.0-149.0), and the median DSS was 141.9 months (SE 28.8; CI 95% 85.4-198.4). Four of eighteen patients died of their disease (3/18 consequently to relapse, 1/18 due to progression after lack of response to primary chemotherapy). OS was 85.9% at five years and 34.4% at ten years. DSS was 93.8% after five years and 56.3% after ten years. The two parameters that were associated with worsened outcome in our study as well as in the comparator group (the DOESAK registry) were development of metastasis or relapse and were significantly associated with survival (Table 2). In our study group, differences between patients who had metastases or suffered from a recurrence and those without an event were significant according to the log-rank test (Mantel-Cox; *p-*value 0.033 and 0.037, respectively). Neither previous irradiation nor the presence of benign lesions, both known risk factors for OSA development 28, were associated with DSS (*p*=0.083). MAGE-A expression was detected in 28% of the OSAJ patients (5/18). Neoadjuvant chemotherapy did not affect MAGE-A expression (4/18), and no significant association of MAGE-A expression with survival was found (*p*=0.569).

Univariate analysis using the Cox proportional hazard model revealed no further significant risk factors (data not shown). As a consequence, we did not include additional putative prognostic variables in the multivariate model.

## DISCUSSION

Here, we describe for the first time the institutional results of two decades of treatment for OSAJ. As mentioned above, OSAJ is a rare disease. Different treatment approaches and diverse treatment modalities, as well as other factors, therefore characterize the current state of treatment for this type of sarcoma. In this study, we present a retrospectively analyzed group of patients with OSAJs treated at a single institute and compared our data with the two largest published datasets of OSAJs 1,3. In rare cases, an OSA may also be induced secondarily after radiation therapy. Most of the knowledge concerning radiation-induced OSAJs is derived from case reports 4,5. We identified two cases of potentially induced sarcoma in our collective, and we have provided preliminary evidence for a worsened prognosis of these tumors. We also investigated the potential role of MAGE-A expression as a prognostic marker.

As OSAJ is primarily considered a localized disease, radical resection is the preferred treatment modality 8,29. Surgery was performed in 90% of our patients, a frequency similar (97%) to that reported in the DOESAK collective 1, as well as to the percentage of operations (81.6%) reported by Lee et al. 3. In the retrospective cohort analysis of the SEER cancer registry conducted by Lee et al. 3, 59% of the patients were operated, and 22.6% of the patients had surgery plus radiation therapy, while in 10.7% of the cases, no treatment was performed. In our study, no surgery was performed in 11% of OSAJ patients because of the presence of unresectable tumors at the time of diagnosis. In the collective published by Baumhoer et al. 1, 2.3% of OSAJ cases were reported to be unresectable.

DSS after 10 years in our study group (56%) was comparable to the DSS in the DOESAK registry group (59% 1) as well as in the SEER database cohort (54% 3). The median OS in the present study (10 months) was similar to the OS (8 months) described by Lee et al. 3. Unfortunately, Lee et al. 3 did not provide information about the impact of surgery alone on DSS, although surgery was the only significant parameter of the study collective that affected survival when compared to the DOESAK registry 1 and the SEER tumor database 3. Lee et al. 3 did not report on the incidence of recurrence, and they did not provide follow-up data or results about the incidence or rate of metastasis in their analysis. When comparing our 5-year and 10-year DSS data with those of Baumhoer et al. 1 (Table 2), metastatic and recurrent disease also significantly influenced DSS (*p-*values 0.033 and 0.037). In our study, the combination of surgery with chemotherapy had no significant impact on DSS (*p*=0.619), and no significant association was found between MAGE-A expression and DSS (*p*=0.569).

Most OSAJs were high-grade tumors of the osteoblastic subtype (78%), similar to the incidence reported (75%) in the DOESAK registry 1 and the study (73%) by Lee et al. 3. Seventeen percent of the high-grade tumors had a chondroblastic subtype, compared to 16% 1 and 20% 3 in the comparative datasets. The third most frequent subtype, the fibroblastic subtype, showed a similar prevalence in the three analyzed collectives (present study: 5%, DOESAK: 9% 1, SEER: 7% 3). We found a tendency in our collective towards a more frequent application of adjuvant chemotherapy (39% in our collective *versus* 14% in the DOESAK registry group 1). No comparison was possible with the data from Lee et al. 3, who did not provide any information about chemotherapy in combination with surgery. However, the addition of chemotherapy was not associated with an improvement in the time to development of metastasis (median 23.8 months with chemotherapy *versus* 26 months without chemotherapy). A local recurrence rate of 33% in our collective was similar to the percentage (44%) reported by Baumhoer et al. 1. The average time to local recurrence in the DOESAK registry cohort 1 was 22.5 months, which is comparable to the mean time to relapse of 18.17 months in the study collective.

Radiation-induced OSAs are considered highly aggressive lesions with local recurrence rates of up to 86% in comparison to 22% of primary OSAs of the head and neck 30,31. Although radiation-induced OSAs are not explicitly reported in the study by Baumhoer et al. 1, we identified two patients who had previous radiotherapy more than 15 years before the occurrence of the sarcoma. One patient was treated for a benign lesion of the ethmoid sinus with a total dose of 53 Gy, and one patient was irradiated for a nasopharyngeal squamous cell carcinoma with a total dose of 70 Gy. The other two patients with risk factors had fibrous dysplasia in their medical history 32,33.

We hypothesized that the majority of OSAJ patients would be MAGE-A negative, as OSAJs are described in the literature to be more “benign” than OSAPs are. Indeed, we found no statistically significant association of MAGE-A expression with survival.

This study has some obvious limitations, such as its small number of patients and its retrospective nature, and conclusions based on these data have to be taken with caution. However, our collective, with respect to some fundamental basic characteristics, such as age distribution, outcome data, and histology, seems to represent quite well the patient collectives from other institutions. Our study collective has similar demographics as well as tumor-associated characteristics compared to those of the two reference groups, except for the utilization of the treatment modalities (Tables 1 and 2). The age distribution (mean 42 years) of the presented patient cohort resembled the expected distribution (mean of 39 years in the DOESAK registry 1) and the published mean of 41 years in the literature 3,34. In two-thirds of the patients, the mandible was the primary affected site. A similar preference for the mandible as the primary site of occurrence was reported in the study by Baumhoer et al. 1, while Lee et al. 3 described a 50% prevalence for the lower jaw. In contrast to our study, neither Lee et al. 3 nor Baumhoer et al. 1 differentiated between maxillary OSAJs and craniofacial OSAs, which are known to show a significantly worse prognosis 9,35.

Lee et al. 3 mentioned several limitations of the SEER database, such as the ambiguous anatomical assignment of tumors to the maxilla or other craniofacial sites. Additionally, the majority of OSAJs that were classified as “not otherwise specified” (NOS) were automatically regarded as tumors of the osteoblastic subtype. In our view, because of such uncertainties in large databases, single-institutional reports, despite small patient numbers, may have some distinct advantages.

In conclusion, prospective studies of OSAJs are only feasible in a multicenter study setting and need to be conducted over prolonged time periods. Therefore, uncertainties about the optimal treatment strategies will most likely remain as factors that may affect the outcome of OSAJ patients and are reported differently in the literature 1,3,36.

## AUTHOR CONTRIBUTIONS

Eder-Czembirek C and Selzer E conceived and designed the study and were responsible for the data acquisition, manuscript preparation, editing, reviewing. Moser D and Sulzbacher I were responsible for the data acquisition, data quality control, data analysis and interpretation, and manuscript reviewing. Holawe S was responsible for the data acquisition, analysis, interpretation and manuscript reviewing. Brodowicz T was responsible for the quality control of data and algorithms, analysis, interpretation and manuscript reviewing. Ries J was responsible for the quality control of data and algorithms, and manuscript reviewing.

## Figures and Tables

**Table 1:** Demographic, tumor and treatment characteristics. Data were compared with data from the DOESAK registry (1) and the SEER tumor database (3). For comparisons of the means between the groups, Fisher’s test (two-tailed) was used.

	Study group N=18* N (%)	DOESAK N=214* N (%)	*p-*value	SEER N=541* N (%)	*p-*value
Age (years)					
Mean (SD)	42 (16)	39 (19)	0.516	41 (21)	0.842
Range	24-80	3-83		0-90	
Sex					
Female	7 (39)	104 (49)	0.471	271 (50)	0.474
Male	11 (61)	110 (51)		270 (50)	
Location					
Maxilla	6 (33)	78 (36)	1.000	301 (56)	0.090
Mandible	12 (67)	136 (64)		240 (44)	
Surgery		N = 201		N = 533	
No resection	2 (11)	5 (2)	0.105	79 (16)	1.000
Resection	16 (89)	196 (98)		454 (84)	
Tumor grade				N = 327	
High grade	18 (100)	197 (92)	0.007	221 (68)	<0.0001
Low grade	0 (0)	17 (8)		106 (32)	
Histological subtype**		N = 192		N = 507	
Osteoblastic	14 (78)	144 (75)	1.000	372 (73)	1.000
Chondroblastic	3 (17)	31 (16)		102 (20)	
Fibroblastic	1 (5)	17 (9)		33 (7)	
Primary treatment modality	N=16	N=179		N=442	
Surgery alone	4 (25)	127 (71)	0.0004	320 (72)	0.0002
Surgery & CHT or RT	12 (75)	52 (29)		122 (28)	

*N=18 in the study group; N=214 in the DOESAK and N=541 in the SEER collectives if not indicated otherwise.

**Only the most common high-grade histological subtypes are shown.

**Table 2:** Study group *versus* the DOESAK registry group (1). Five- and 10-year disease-specific survival rates (%) according to different variables.

Parameter	Subgroup	Study group			DOESAK registry group		
		5 y	10 y	*P*_LR-test_	5 y	10 y	*P*_LR-test_
Gender	Male	87.5	43.8	0.164	66.5	61.0	0.961
	Female	83.3	0		66.9	56.5	
Origin	Mandible	90.9	68.2	0.751	66.8	59.9	0.811
	Maxilla	100	0		66.6	58.7	
Tumor grade	Low	na	na	na	100	100	0.027
	High	93.8	56.3		64.6	56.7	
Histological subtype	Osteoblastic	88.9	29.6	0.240	63.8	58.0	0.096
	Chondroblastic	0	0		75.7	63.1	
Metastases	No	100	na	0.033	78.3	74.1	<0.001
	Yes	80.0	26.7		19.7	7.9	
Recurrence	No	100	60.0	0.037	83.2	83.2	<0.001
	Yes	66.7	na		51.9	48.7	

na: not available.
